# 

**Published:** 1914-04-25

**Authors:** 


### CONTENTS.

PAGE

**The Government of the Royal College of Surgeons** ... ... ... ... ... ... 85

**Good Work Done and More to Follow at Leicester** ... ... ... ... ... ... 86

**Hospital and Institutional News** ... ... ... 87

The King and a Paris Hospital—The Doctor's Wives Association—Funeral of Mr. Sydney Phillips—Administrative Promotions in Sanatoria—Effect of Specialism on Infirmary Work—Two Criticisms of the Bristol Scheme—A Scare for Hospital Bibliographers—Doctors, Disease. and the Law of Libel—A New General Hospital for Lincolnshire—"An Insanitary and Obscure Village"—Baths for Continuous Immersion—The Hen Out-Patient—The Hen and Hospital Economy—Operative Work in a London Infirmary—Isolation Hospital Officer Vindicated—A Fire in a Doctor's Surgery—A Chaplaincy Departure at Greenwich—The Dog and Research—The Insurance Act and Infirmary Confinements—Infirmaries and the Abuse of Benefits—A Bequest to Sanatorium Convalescents—The Medical Staff of the City of Westminster Infirmary—Christianity and Medicine in China—Whale Oil as a New Food—New Assistant Secretary at Cardiff—Mexico and the Drug Market—To Our Readers.

PAGE

**Modern Methods Series** ... ... ... ... 3

The "Cardiac Sign" of Malignant Disease.

**Clinics for Mental Disease** ... ... ... ... ... 94

Their Desirability in General Hospitals.

**Clubbed Fingers in Children** ... ... ... 95

An Aid to Prognosis for the School Doctor.

**The Picture Palace and the Spread of Disease** 96

**Reports on Hospitals of the United Kingdom**— 97

By SIR HENRY BURDETT, K.C.B., K.C.V.O

The Ashford Cottage Hospital.

**The Complexity of Diphtheria Toxin** ... ... 98

**National Insurance Act Developments** ... ... 99

The Future of the Deposit Contributor.

**Round the World** ... ... ... ... ... 101

Fellow-Workers and their Doings.

**The Patients' Column** ... ... ... 1[illegible]2

The Diary of a "Chronic Appendix."

[*See over.*]

### CONTENTS—(*continued*).

| | PAGE |
|---|---|
| **Appointments** ... ... ... ... ... ... ... | 102 |
| **Personalia** ... ... ... ... ... ... ... | 102 |
| **The First Hospital in the British Solomon Islands** ... ... ... ... ... ... ... | 103 |
| The Welchman Memorial Hospital, Guadalcanar. | |
| **The Late Mr. Sydney Phillips** ... ... ... | 104 |
| His Work and his Personality. | |
| **The Editor's Letter-Box** ... ... ... ... | 105 |
| Non-Panel Men and the "Medical Directory"—Women Doctors and the London County Council—A Unique Hospital Chapel. | |
| **The Right Treatment of Legacies** ... ... | 107 |
| Their Place in the Hospital Budget. | |
| **The Doctors' Wives Association** ... ... ... | 108 |
| **The Medical Officer's Library** ... ... ... | 109 |
| The Barometer of Medical Progress—A New Text-Book on Surgery—Still upon Goodhart—The Pocket Guide Series. | |
| **Doctors' Wills** ... ... ... ... ... ... ... | 110 |
| **Gifts and Bequests** ... ... ... ... ... | 110 |
| **Institutional Needs** ... ... ... ... ... | 110 |
| **The National Medical Union (Official)** ... ... | 111 |
| Dr. Macevoy on the Constitution. | |
| The Hampstead Non-Panel Association and the Registrar. | |
| Sir John Collie on State Medical Service. | |
| **The Institutional Worker** ... ... (Supp l.) | 1 |
| The Chaplain's Department. | |
| Questions for April. | |
| Questions Invited. | |
| Caring for Poor Babies in Leeds. | |
| The Sick and in Need. | |
| Employment and Training. | |
| **The Book Market** ... ... ... ... ,, | 3 |
| **Buildings Contemplated** ... ... ... ,, | 3 |

